# Outpatient non-operative treatment of uncomplicated acute appendicitis: meta-analysis

**DOI:** 10.1093/bjsopen/zrag138

**Published:** 2026-09-10

**Authors:** Mauro Podda, Adolfo Pisanu, Fausto Catena, Marco Ceresoli

**Affiliations:** Department of Surgical Science, University of Cagliari, Cagliari, Italy; Department of Surgical Science, University of Cagliari, Cagliari, Italy; Department of Medical and Surgical Sciences (DIMEC), Alma Mater Studiorum, University of Bologna, Bologna, Italy; School of Medicine and Surgery, University of Milano-Bicocca, Monza, Italy

**Keywords:** conservative treatment, outpatient management, antibiotic therapy, systematic review

## Abstract

This systematic review and meta-analysis compared outpatient versus in-hospital antibiotic management for imaging-confirmed uncomplicated acute appendicitis. Three comparative studies including 1 064 patients found that outpatient treatment was associated with a lower 30-day appendicectomy rate, with similar overall complications and unplanned healthcare use. Although certainty of evidence was low to very low, outpatient antibiotic management may be feasible for appropriately selected patients within structured pathways ensuring diagnostic certainty, follow-up, and rapid access to reassessment.

Although antibiotic-first treatment is now an accepted option for select patients with imaging-confirmed uncomplicated appendicitis, whether hospital admission is required remains uncertain^1^.

This systematic review and meta-analysis was performed in accordance with PRISMA guidelines to compare outpatient *versus* in-hospital non-operative management with antibiotics for imaging-confirmed uncomplicated acute appendicitis. Detailed methods are reported in PROSPERO (CRD420261391790). Comparative studies, including secondary analyses of randomized trials, were eligible if outpatient management was defined as discharge home within 24 hours (h) of hospital presentation. The co-primary outcomes were appendicectomy and overall complications within 30 days. Secondary outcomes included 30-day unplanned healthcare use and cumulative length of hospital stay. Risk of bias was assessed using the ROBINS-I tool (www.riskofbias.info). Certainty of evidence was assessed using the GRADE approach (www.gradepro.org). Statistical analyses were performed using Cochrane RevMan Web.

Three studies were included (two observational studies and one secondary analysis of the CODA randomized trial), comprising 1064 patients. Two were adult studies and one was a paediatric study, with 502 patients managed as outpatients and 562 managed in hospital (*Supplementary material*). Outpatient treatment was associated with a lower 30-day appendicectomy rate (3 studies, 1064 patients; odds ratio (OR) 0.52; 95% confidence interval (c.i.) 0.36 to 0.76; *I^2^* = 0%). Overall complications and 30-day unplanned healthcare use were similar between groups (*Fig. 1*). The CODA secondary analysis reported serious adverse events at 7 days in 0.9 per 100 participants discharged within 24 h and in 1.3 per 100 participants discharged after 24 h. At 30 days, serious adverse events occurred in 1.8 per 100 outpatients and in 3.6 per 100 inpatients^2^.

**Figure 1:**
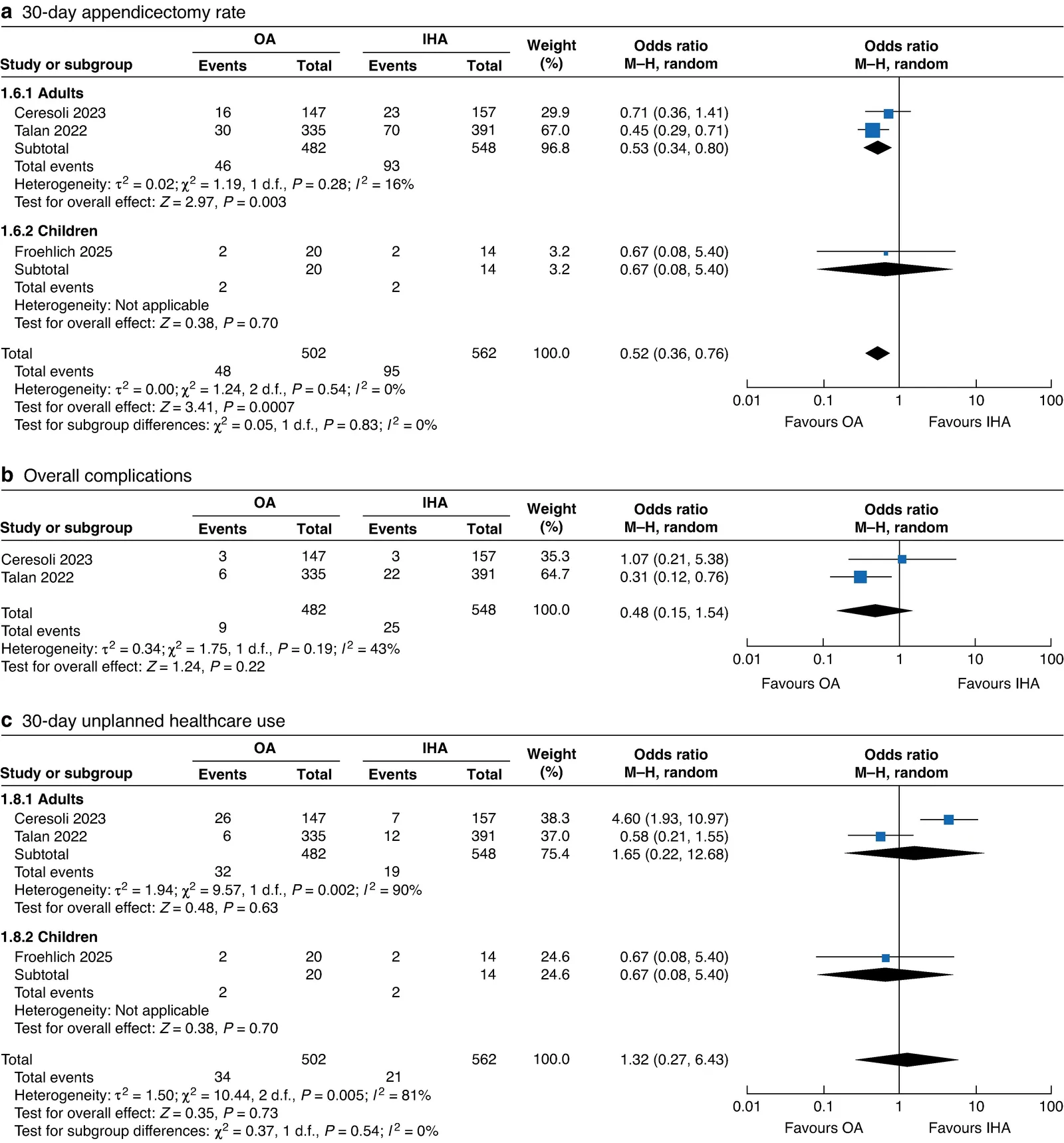
Forest plots of clinical outcomes **a** Thirty-day appendicectomy rate, **b** overall complications, and **c** 30-day unplanned healthcare use comparing OA *versus* IHA. Values in parentheses are 95% confidence intervals. OA, outpatient antibiotics; IHA, in-hospital antibiotics; M-H, Mantel–Haenszel.

In the subgroup analysis restricted to adult studies, outpatient management was associated with a lower 30-day appendicectomy rate (2 studies, 1030 patients; OR 0.53; 95% c.i. 0.34 to 0.80; *I^2^* = 16%), whereas no significant difference was observed in 30-day unplanned hospital admission. In the CODA secondary analysis, health-related quality of life at 30 days was similar between outpatient and in-hospital treatment (mean(standard deviation) EQ-5D™ 0.93(0.003) *versus* 0.92(0.005), respectively). Treatment dissatisfaction at 7 days was lower in the outpatient group (7.3% *versus* 9.7%)^2^. Two studies reported cumulative length of hospital stay, which was shorter with outpatient management (0.89 *versus* 3.94 days in the study of Ceresoli *et al.*; 0.29 *versus* 0.95 days in the study of Froehlich *et al.*). Results are presented descriptively because of marked between-study heterogeneity. ROBINS-I rated all included studies at overall serious risk of bias. GRADE assessment rated the certainty of evidence as low to very low across all critical outcomes (*Supplementary material*).

Viewed against the current evidence, antibiotic-first treatment is an accepted option for select patients with imaging-confirmed uncomplicated appendicitis. The remaining question is whether all such patients require admission, or whether some components of care can safely be delivered through structured outpatient pathways. Previous antibiotics *versus* appendicectomy trials provide context. In the 2017 US pilot trial by Talan *et al.*^3^, 14 of 15 antibiotic-treated adults were discharged from the emergency department and all had symptom resolution. In APPAC II^4^, oral moxifloxacin achieved 1-year treatment success in 70.2% of patients. In CODA^2^, 46.1% of patients treated with antibiotics alone were discharged within 24 h; outpatients missed fewer workdays, with similar return visits, satisfaction, and EQ-5D™ scores. The APPAC III trial^5^ reported 10-day success in 87% of patients with placebo and in 97% of patients with antibiotics, without increased perforation.

Outpatient antibiotic management may be feasible for appropriately selected patients with uncomplicated appendicitis within experienced pathways ensuring diagnostic certainty, structured follow-up, and rapid access to reassessment.

## Supplementary Material

zrag138_Supplementary_Data

## Data Availability

The data, analytical methods, and materials used in this study are available in the Supplementary material. No custom code was used beyond standard functions implemented in Cochrane Review Manager.
