# Supplementary material for: Outpatient non-operative treatment of uncomplicated acute appendicitis: meta-analysis
Source: BJS Open. 2026 Sep 10;10(5):zrag138. doi: 10.1093/bjsopen/zrag138 (PMC13560070; doi:10.1093/bjsopen/zrag138)
Supplement: Supplementary file 1 [file zrag138_supplementary_data.docx]

**Outpatient Non-operative Treatment of Uncomplicated Acute Appendicitis:**

**Short Systematic Review and Meta-analysis.**

Mauro Podda MD^1^, Adolfo Pisanu MD PhD^1^, Fausto Catena MD PhD^2^, Marco Ceresoli MD^3^.

1. Department of Surgical Science, University of Cagliari, Cagliari, Italy.
2. Department of Medical and Surgical Sciences (DIMEC), Alma Mater Studiorum, University of Bologna, Bologna, Italy.
3. University of Milano-Bicocca, School of Medicine and Surgery, Monza, Italy.

**Corresponding Author**

Prof. Mauro Podda MD, FACS

Professor of Surgery

Department of Surgical Science

University of Cagliari

Cagliari, Italy.

[mauro.podda@unica.it](mailto:mauro.podda@unica.it)

ORCID ID: 0000-0001-9941-0883

**Supplementary Tables & Figures**

**Supplementary Table S1.** General characteristics of the studies included in the systematic review & meta-analysis. **Page 3.**

**Supplementary Table S2.** Clinical outcomes. **Page 4.**

**Supplementary Table S3.** Patient-reported outcomes. **Page 5.**

**Supplementary Table S4.** Results of the meta-analysis. **Page 6.**

**Supplementary Figure S1.** PRISMA flow diagram of study selection. Identification, screening, eligibility, and inclusion of studies in the systematic review and meta-analysis. **Page 7.**

**Supplementary Figure S2.** Risk of bias assessment. Risk of bias for non-randomised comparative studies (ROBINS-I tool). **Page 8.**

**Supplementary Figure S3.** GRADE summary of findings showing the certainty of evidence for the critical outcomes. **Page 9.**

**References**

Ceresoli M, Fumagalli C, Fugazzola P, Zanini N, Magnone S, Ravasi M, Bonalumi J, Morezzi D, Bova R, Sargenti B, Schiavone L, Lucianetti A, Catena F, Ansaloni L, Braga M. Outpatient Non-operative Management of Uncomplicated Acute Appendicitis: A Non-inferiority Study. World J Surg 2023;47:2378-2385. doi: 10.1007/s00268-023-07065-7.

Writing Group for the CODA Collaborative; Talan DA, Moran GJ, Krishnadasan A, Monsell SE, Faine BA, Uribe L, Kaji AH, DeUgarte DA, Self WH, Shapiro NI, Cuschieri J, Glaser J, Park PK, Price TP, Siparsky N, Sanchez SE, Machado-Aranda DA, Victory J, Ayoung-Chee P, Chiang W, Corsa J, Evans HL, Ferrigno L, Garcia L, Hatch Q, Horton MD, Johnson J, Jones A, Kao LS, Kelly A, Kim D, Kutcher ME, Liang MK, Maghami N, McGrane K, Minko E, Mohr C, Neufeld M, Patton JH, Rog C, Rushing A, Sabbatini AK, Salzberg M, Thompson CM, Tichter A, Wisler J, Bizzell B, Fannon E, Lawrence SO, Voldal EC, Lavallee DC, Comstock BA, Heagerty PJ, Davidson GH, Flum DR, Kessler LG. Analysis of Outcomes Associated With Outpatient Management of Nonoperatively Treated Patients With Appendicitis. JAMA Netw Open 2022;5(7):e2220039. doi: 10.1001/jamanetworkopen.2022.20039.

Froehlich M, Karunungan K, Jen HC, Wagner JP, Shekherdimian S, Sullins VF, DeUgarte DA, Lee S. Non-Operative Management of Uncomplicated Appendicitis in Children: Can Patients Be Discharged From the Emergency Department? J Pediatr Surg 2025;60:161643. doi: 10.1016/j.jpedsurg.2024.07.028.

**Supplementary Table S1.** General characteristics of the studies included in the systematic review & meta-analysis.

| **Author, Year** | **Study Design** | **Country** | **Care protocols** | **N. Patients Outpatient antibiotics**  **OA**  **N. (%)** | **N. Patients In-hospital antibiotics**  **IHA**  **N. (%)** | **Male sex**  **OA**  **N. (%)** | **Male sex**  **IHA**  **N. (%)** | **Age (years)**  **OA**  **Mean ± SD** | **Age (years)**  **IHA**  **Mean ± SD** | **Days from symptoms onset**  **OA**  **Mean ± SD** | **Days from symptoms onset**  **IHA**  **Mean ± SD** | **Appendicolith OA**  **N. (%)** | **Appendicolith**  **IHA**  **N. (%)** | **Appendiceal diameter on imaging (mm)**  **OA**  **Mean ± SD** | **Appendiceal diameter on imaging (mm)**  **IHA**  **Mean ± SD** |
| --- | --- | --- | --- | --- | --- | --- | --- | --- | --- | --- | --- | --- | --- | --- | --- |
| **Talan 2022** | RCT* | USA | *Outpatient*  Once-daily IV regimen (ertapenem or ceftriaxone and high-dose metronidazole) or scheduled doses with coverage for 24  hours followed by oral antibiotics to complete a 10-day total course.  *In-hospital*  IV formulation for at least 24 hours, followed by pills, for a 10-day total course. | 335 (46.1%) | 391 (53.9%) | 213 (63.6%) | 249 (63.7%) | NR | NR | NR | NR | 86 (25.7%) | 108 (27.6%) | 11 ± 0.16 | 12 ± 0.16 |
| **Ceresoli 2023** | Observational (retrospective) | Italy | *Outpatient*  Patients discharged home  within 12 h from the arrival in the emergency department  received only oral  amoxicillin/clavulanate or ciprofloxacin).  *In-hospital*  In-hospital patients were given intravenous antibiotics during hospitalization  followed by oral antibiotics at home. | 147 (22.1%)** | 157 (23.5) | 75 (51.0%) | 83 (52.9%) | 27 ± 3.66 | 37 ± 5.0 | 1 ± 0.16 | 2 ± 0.16 | 4 (2.7%) | 16 (10.2%) | 9 ± 0.33 | 10 ± 0.5 |
| **Froehlich 2025** | Observational (prospective) | USA | *Outpatient*  A dose of IV antibiotics in the emergency department (ceftriaxone and metronidazole). Patients then completed a course of oral antibiotics (amoxicillin/clavulanic acid for 7 days).  *In-hospital*  Not specified. | 20 (59.0%) | 14 (41.0%) | NR | NR | 12 ± 2.8 | 10 ± 3.6 | 1.0 ± 0.58 | 1.08 ± 0.75 | NR | NR | NR | NR |

*Secondary analysis; **364 (54.3%) patients were given upfront appendectomy, 157 (23.5%) patients were admitted for in-hospital NOM, and 147 (22.1%) patients were discharged home for outpatient NOM; NR= Not Reported; SD= Standard Deviation.

**Supplementary Table S2.** Clinical outcomes.

| **Author, Year** | **Appendicectomy OA**  **N. (%)** | **Appendicectomy**  **IHA**  **N. (%)** | **Overall complications OA**  **N. (%)** | **Overall complications**  **IHA**  **N. (%)** | **Serious adverse events**  **OA**  **N. (%)*** | **Serious adverse events**  **IHA**  **N. (%)*** | **Complicated appendicitis at surgery**  **OA**  **N. (%)** | **Complicated appendicitis at surgery**  **IHA**  **N. (%)** | **Unplanned healthcare use OA**  **N. (%)** | **Unplanned healthcare use**  **N. (%)** | **Missed workdays (days) OA**  **Mean ± SD** | **Missed workdays (days) IHA**  **Mean ± SD** |
| --- | --- | --- | --- | --- | --- | --- | --- | --- | --- | --- | --- | --- |
| **Talan 2022** | 33 (9.9%) 7 days  30 (12.6%) 30 days  64 (19.0%)  7 days (appendicolith) | 55 (14.1%) 7 days  70 (19.0%) 30 days  91 (23.4%)  7 days  (appendicolith) | 6 (1.8%)  7 days | 22 (5.6%)  7 days | 3 (0.9%)  7 days  6 (1.8%) 30 days | 5 (1.3%)  7 days  14 (3.6%) 30 days | NR | NR | 3 (0.9%)  7 days  6 (1.8%)  30 days | 4 (1.0%)  7 days  12 (3.1%)  30 days | 2.6 ± 0.10  7 days | 3.8 ± 0.15  7 days |
| **Ceresoli 2023** | 16 (10.9%)  30 days | 23 (14.6%)  30 days | 3 (2.0%) | 3 (1.9%) | - | - | 5 (3.4%) | 3 (1.9%) | 26 (17.7%)  30 days | 7 (4.4%)  30 days | NR | NR |
| **Froehlich 2025** | 2 (10%)  30 days | 2 (14.3%)  30 days | - | - | - | - | - | - | 2 (10%)  30 days | 2 (14.3%)  30 days | NA | NA |

*Serious adverse event was defined as death, life-threatening event, significant treatment-related disability or incapacity, or non–appendicitis related hospitalization; NR= Not Reported; NA= Not Applicable; SD= Standard Deviation.

**Supplementary Table S3.** Patient-reported outcomes.

| **Author, Year** | **Cumulative LOS (days)**  **OA**  **Mean ± SD** | **Cumulative LOS (days)**  **IHA**  **Mean ± SD** | **Quality of life**  **OA**  **Mean ± SD** | **Quality of life**  **IHA**  **Mean ± SD** | **Treatment dissatisfaction**  **OA**  **N. (%)** | **Treatment dissatisfaction**  **IHA**  **N. (%)** |
| --- | --- | --- | --- | --- | --- | --- |
| **Talan 2022** | NR | NR | 0.93 ± 0.003*  30 days | 0.92 ± 0.005*  30 days | 22 (7.3%)  7 days | 32 (9.7%)  7 days |
| **Ceresoli 2023** | 0.89 ± 1.94 | 3.94 ± 2.17 | NR | NR | NR | NR |
| **Froehlich 2025** | 0.29 ± 0.12 | 0.95 ± 0.45 | NR | NR | NR | NR |

*Numbers for EQ-5D are 306 for time to discharge of 24 hours or less, 337 for time to discharge of greater than 24 hours; NR= Not Reported; SD= Standard Deviation.

**Supplementary Table S4.** Results of the meta-analysis.

| **Variable** | **Studies** | **Participants** | **Statistical Method** | **Effect Estimate** | **Heterogeneity** |
| --- | --- | --- | --- | --- | --- |
| **Male sex (Adults)** | 2 | 1030 | Odds Ratio (M-H, Random, 95% CI) | 0.97 [0.76, 1.25] | *I^2^* = 0% |
| **Age** | 2 | 338 | Mean Difference (IV, Random, 95% CI) | -4.04 [-15.80, 7.71] | *I^2^* = 99% |
| **Age (Children)** | 1 | 34 | Mean Difference (IV, Random, 95% CI) | 2.00 [-0.25, 4.25] | - |
| **Age (Adults)** | 1 | 304 | Mean Difference (IV, Random, 95% CI) | -10.00 [-10.98, -9.02] | - |
| **Days from symptoms onset** | 2 | 338 | Mean Difference (IV, Random, 95% CI) | -0.57 [-1.47, 0.33] | *I^2^* = 93% |
| **Days from symptoms onset (Children)** | 1 | 34 | Mean Difference (IV, Random, 95% CI) | -1.00 [-1.04, -0.96] | - |
| **Days from symptoms onset (Adults)** | 1 | 304 | Mean Difference (IV, Random, 95% CI) | -0.08 [-0.55, 0.39] | - |
| **Appendicolith (Adults)** | 2 | 1030 | Odds Ratio (M-H, Random, 95% CI) | 0.53 [0.15, 1.86] | *I^2^* = 79% |
| **Appendiceal diameter (Adults)** | 2 | 1030 | Mean Difference (IV, Random, 95% CI) | -1.00 [-1.02, -0.98] | *I^2^* = 0% |
| **30-day appendicectomy** | 3 | 1064 | Odds Ratio (M-H, Random, 95% CI) | 0.52 [0.36, 0.76] | *I^2^* = 0% |
| **30-day appendicectomy (Children)** | 1 | 34 | Odds Ratio (M-H, Random, 95% CI) | 0.53 [0.34, 0.80] | - |
| **30-day appendicectomy (Adults)** | 2 | 1030 | Odds Ratio (M-H, Random, 95% CI) | 0.67 [0.08, 5.40] | *I^2^* = 16% |
| **Overall complications (Adults)** | 2 | 1030 | Odds Ratio (M-H, Random, 95% CI) | 0.48 [0.15, 1.54] | *I^2^* = 43% |
| **30-day unplanned hospital admission** | 3 | 1064 | Odds Ratio (M-H, Random, 95% CI) | 1.32 [0.27, 6.43] | *I^2^* = 81% |
| **30-day unplanned hospital admission (Children)** | 1 | 34 | Odds Ratio (M-H, Random, 95% CI) | 1.65 [0.22, 12.68] | - |
| **30-day unplanned hospital admission (Adults)** | 2 | 1030 | Odds Ratio (M-H, Random, 95% CI) | 0.67 [0.08, 5.40] | *I^2^* = 90% |
| **30-day recurrent appendicitis** | 2 | 760 | Odds Ratio (M-H, Random, 95% CI) | 0.62 [0.41, 0.92] | *I^2^* = 0% |
| **30-day recurrent appendicitis (Children)** | 1 | 34 | Odds Ratio (M-H, Random, 95% CI) | 0.61 [0.41, 0.93] | - |
| **30-day recurrent appendicitis (Adults)** | 1 | 726 | Odds Ratio (M-H, Random, 95% CI) | 0.67 [0.08, 5.40] | - |
| **Cumulative length of hospital stay** | 2 | 338 | Mean Difference (IV, Random, 95% CI) | -1.85 [-4.19, 0.50] | *I^2^* = 99% |
| **Cumulative length of hospital stay (Children)** | 1 | 34 | Mean Difference (IV, Random, 95% CI) | -3.05 [-3.51, -2.59] | - |
| **Cumulative length of hospital stay (Adults)** | 1 | 304 | Mean Difference (IV, Random, 95% CI) | -0.66 [-0.90, -0.42] | - |

**Supplementary Figure S1.** PRISMA flow diagram of study selection. Identification, screening, eligibility, and inclusion of studies in the systematic review and meta-analysis.

**Supplementary Figure S2.** Risk of bias assessment. Risk of bias for non-randomised comparative studies (ROBINS-I tool).

**Supplementary Figure S3.** GRADE summary of findings showing the certainty of evidence for the critical outcomes.
